# Spillover of ebolaviruses into people in eastern Democratic Republic of Congo prior to the 2018 Ebola virus disease outbreak

**DOI:** 10.1186/s42522-020-00028-1

**Published:** 2020-11-04

**Authors:** Tracey Goldstein, Manjunatha N. Belaganahalli, Eddy K. Syaluha, Jean-Paul K. Lukusa, Denise J. Greig, Simon J. Anthony, Alexandre Tremeau-Bravard, Riddhi Thakkar, Adrian Caciula, Nischay Mishra, W. Ian Lipkin, Jasjeet K. Dhanota, Brett R. Smith, Victoria M. Ontiveros, Nistara Randhawa, Michael Cranfield, Christine K. Johnson, Kirsten V. Gilardi, Jonna A. K. Mazet

**Affiliations:** 1 One Health Institute & Karen C Drayer Wildlife Health Center, School of Veterinary Medicine, University of California Davis, California, USA; 2 Mountain Gorilla Veterinary Project Inc, Goma, Democratic Republic of the Congo; 3 Center for Infection and Immunity, Mailman School of Public Health, Columbia University, 722 West 168th Street, New York, NY 10032 USA; 4 Department of Epidemiology, Mailman School of Public Health, Columbia University, 722 West 168th Street, New York, NY USA

**Keywords:** Ebola virus, Bombali virus, Ebola virus disease, Ebolavirus serology, Eastern DRC, Zoonosis

## Abstract

**Background:**

The second largest Ebola virus disease (EVD) outbreak began in the Democratic Republic of Congo in July 2018 in North Kivu Province. Data suggest the outbreak is not epidemiologically linked to the 2018 outbreak in Equateur Province, and that independent introduction of Ebola virus (EBOV) into humans occurred. We tested for antibodies to ebolaviruses in febrile patients seeking care in North Kivu Province prior to the EVD outbreak.

**Methods:**

Patients were enrolled between May 2017 and April 2018, before the declared start of the outbreak in eastern DRC. Questionnaires were administered to collect demographic and behavioural information to identify risk factors for exposure. Biological samples were evaluated for ebolavirus nucleic acid, and for antibodies to ebolaviruses. Prevalence of exposure was calculated, and demographic factors evaluated for associations with ebolavirus serostatus.

**Results:**

Samples were collected and tested from 272 people seeking care in the Rutshuru Health Zone in North Kivu Province. All patients were negative for filoviruses by PCR. Intial screening by indirect ELISA found that 30 people were reactive to EBOV-rGP. Results were supported by detection of ebolavirus reactive linear peptides using the Serochip platform. Differential screening of all reactive serum samples against the rGP of all six ebolaviruses and Marburg virus (MARV) showed that 29 people exhibited the strongest reactivity to EBOV and one to Bombali virus (BOMV), and western blotting confirmed results. Titers ranged from 1:100 to 1:12,800. Although both sexes and all ages tested positive for antibodies, women were significantly more likely to be positive and the majority of positives were in February 2018.

**Conclusions:**

We provide the first documented evidence of exposure to Ebola virus in people in eastern DRC. We detected antibodies to EBOV in 10% of febrile patients seeking healthcare prior to the declaration of the 2018–2020 outbreak, suggesting early cases may have been missed or exposure ocurred without associated illness. We also report the first known detection of antibodies to BOMV, previously detected in bats in West and East Africa, and show that human exposure to BOMV has occurred. Our data suggest human exposure to ebolaviruses may be more frequent and geographically widespread.

## Background

**Fig. 1:**
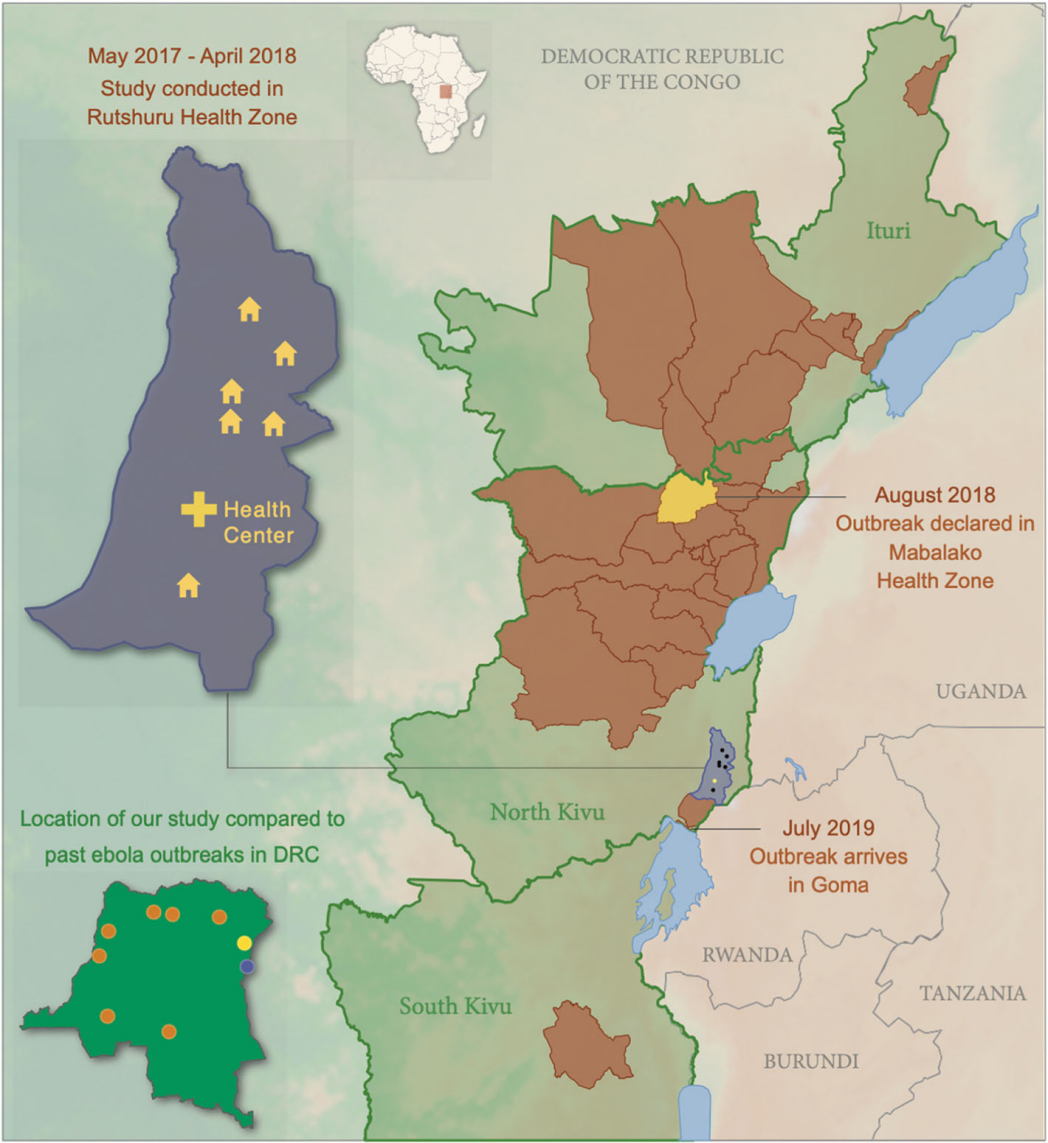
Location of villages and the Rubare Health Center in Rutshuru Health Zone, North Kivu Province where febrile study participants traveled from and were treated prior to Ebola Virus Disease outbreak in Eastern DRC that began in 2018. The outbreak was first observed in the Mabalako Health Zone (yellow) in North Kivu before spreading to other areas (brown). Inset map of the DRC: Location of the 2018–2020 outbreak (yellow), study location (purple) and other EBOV outbreaks in the DRC to date (orange)

The second largest Ebola virus disease (EVD) outbreak was declared in eastern Democratic Republic of Congo (DRC) in August of 2018 following reports of cases in late July, but cases may have occurred as early as April 2018 [1]. Although the index case was not identified, the outbreak is believed to have begun in the village of Mangina in the Mabalako Health Zone in North Kivu Province, quickly spreading to the neighboring Ituri Province and then to South Kivu Province, reaching the most populous city of Goma in July 2019 (Fig. 1). Cases ultimately occurred in 29 health zones [1], and when the end of the outbreak was declared in June 2020 a total of 3481 cases and 2299 deaths were reported. Since the first EVD outbreak in the DRC in Kikwit in 1976, there have been eight outbreaks caused by Ebola virus (EBOV, species *Zaire ebolavirus*) in the DRC [2], but the current outbreak is the first reported outbreak in the eastern region of the country. There have been Ebola disease (EBOD) [3] outbreaks to the north in the Isiro and Dungu Health Zones of Province Orientale in eastern DRC and across the border in Uganda, including in the Bundibugyo and Kibale districts adjacent to North Kivu, but those were caused by Bundibugyo (BDBV, species *Bundibugyo ebolavirus*) and Sudan (SUDV, species *Sudan ebolavirus*) viruses [2, 4, 5]. The current outbreak is not epidemiologically linked to the EVD outbreak that occurred in the Bikoro Health Zone of Equateur Province, DRC from May to August 2018 (more than 780 miles away), and viral genome sequences confirm that the isolate that circulated in eastern DRC was phylogenetically distinct from the other known EBOV strains, suggesting an independent introduction into people. Phylogenetic analysis suggests that the virus may have been circulating in its natural reservoir 1–2 years before the current outbreak began [6].

Ebolaviruses belong to the genus *Ebolavirus* (family *Filoviridae*) and can cause severe and fatal hemorrhagic disease in humans and non-human primates [7]. They are non-segmented, negative-sense, single-stranded RNA viruses that code for seven distinct viral proteins, and the genus contains six recognized species EBOV, SUDV, BDBV, Taï Forest (TAFV, species *Taï Forest ebolavirus*), Reston (RESTV, species *Reston ebolavirus* (RESTV), and Bombali (BOMV, species *Bombali ebolavirus*) [3, 8–10]. All ebolaviruses except BOMV and RESTV are known to cause clinical disease in people with fatality rates ranging from 25 to 90% [11, 12]. Ebolaviruses are zoonotic pathogens and outbreaks result from spillover into humans from wild animals [13]. Using a surrogate virus (rVSV) encoding the BOMV GP gene, BOMV has been shown to have the potential to infect human cells, but it is unknown if spillover to humans has occurred [8]. Little is known about the natural transmission dynamics or the risk factors for human exposure to ebolaviruses but most evidence suggests that bats are the natural hosts [9, 14]. The lethality of viruses such as EBOV have led many to suggest that asymptomatic infections are rare and that spillover events are infrequent; however, growing evidence suggests that human exposure might be more frequent and geographically widespread than previously recognized. For example, in 2013 Smiley-Evans et al. [15] found between 4 and 8% of people in southwestern Uganda were seroreactive to ebolaviruses; and in 2014, Schoepp et al. [16] reported the presence of ebolavirus reactive antibodies in 8.6% of patients with acute febrile illness in Sierra Leone. In both cases, samples were collected from people in regions prior to or where EBOD had not been previously recognized.

Although disease associated with EBOV in North Kivu Province had not been recognized prior to the 2018–2020 outbreak, serologic studies in the DRC and in Uganda suggest that people in the region may have been exposed to ebolaviruses without experiencing clinical disease [15, 17]. North Kivu Province shares porous borders with Uganda and Rwanda where high cross border movement occurs [18], and also includes the Virunga National park (VNP) spanning eastern DRC, Uganda, and Rwanda which has high wild animal biodiversity and where there is close contact between humans and wildlife. Extensive connectivity among communities, poor infrastructure and contact with wildlife may lead to spillover of ebolaviruses into humans. We looked for the presence of antibodies to ebolaviruses in febrile people seeking medical care who lived near the Virunga National Park in North Kivu Province prior to the recent EVD outbreak in that region.

## Methods

### Samples and study sites

Febrile patients seeking care at the Rubare Health Center from the Rutshuru Health Zone, North Kivu Province were included in the study; individuals had travelled from six villages in North Kivu Province: Rubare, Kiwanja Umoja, Biruma, Ntamugemga, and Kalengera (Fig. 1). Patients were enrolled in the study between May 2017 and April 2018, before the documented start of the EVD outbreak that began in July 2018 in eastern DRC. Demographic and behavioural information including age, gender, occupation, medical and travel history, livelihood, and interaction with domestic animals and wildlife were collected through questionnaires administered in local languages. Biological samples including whole blood, feces, and oral swabs were collected and placed into Trizol and viral transport media (VTM). Whole blood collected into serum separator tubes was centrifuged, serum separated, and stored in 0.5 ml aliquots. All samples were frozen immediately after collection in liquid nitrogen and transferred to a − 80 °C freezer in Goma and then shipped to the University of California Davis on dry ice for testing. All research activities were approved by the institutional review boards of the Rutshuru Health Zone and the University of California, Davis.

### Viral screening by PCR

Total RNA was extracted using Direct-Zol RNA columns (Zymo Research Corp), and cDNA transcribed using Superscript III (Invitrogen). Samples were screened for filoviruses using four assays: 1) a nested filovirus ‘family level’ consensus PCR (cPCR) targeting a 680 bp fragment of the filovirus L gene [9], 2) an *Ebolavirus* ‘genus level’ cPCR targeting a 187 bp fragment of the NP gene [19], 3) a real-time PCR specific for the EBOV virus, targeting the L-gene [20], and 4) a real-time PCR specific for the BOMV virus, targeting the L-gene [9]. Samples were also screened using broadly reactive cPCR assays for corona [21, 22], paramyxo [23], flavi [24], and influenza [25] viruses.

### Ebolavirus serology

An indirect ELISA assay was developed to detect antibodies (IgG) to the glycoprotein (GP) of ebolaviruses using recombinant glycoproteins (rGP) commercially available for EBOV (0.25μg/ml R&D systems, Minneapolis, MN USA), SUDV, BDBV, RESTV and MARV viruses (0.25μg/ml, IBT Bioservices, Rockville, MD USA). We synthesized the rGP for BOMV and TAFV (4μg/ml) and produced polyclonal serum raised in a rabbit against the BOMV-rGP. BOMV and TAFV rGP were synthesized without the transmembrane domains (rGPdTM) and expressed in Expi293 cells and purified. We demonstrated reactivity against all seven filoviruses using polyclonal rabbit sera raised against the GP of EBOV (1:2000, eEnzyme LLC, Gathersburg, MD USA), SUDV (1:1000, IBT BioServices, Rockville, MD, USA), BDBV (1:2000, Sino Biological, Wayne, PA USA), TAFV (1:1000, Alpha Diagnostics, San Antonio, TX USA), RESTV (1:2000, Abcam, Cambridge, MA USA), BOMV (1:2000) and Marburg virus (MARV, species *Marburg virus,* 1:2000, IBT BioServices). Despite cross-reaction, each ebolavirus rGP reacted strongest to its homologous antisera, allowing for differentiation (Table 1).

**Table 1:** Indirect ELISA to compare reactivity of recombinant GP proteins from EBOV, SUDV, RESTV, BDBV, TAFV, BOMV and MARV with polyclonal antibodies against all seven viruses (++++ = O.D. > 4.0, +++ = O.D. 3.0–4.0, ++ = O.D. 2.0–3.0, + = O.D. > 0.5–2.0). - = non-reactive. As expected, some cross-reactivity does occur but differential detection of antibodies against a specific virus is possible

	GP Antigen						
Antibody	EBOV	SUDV	RESTV	BDBV	TAFV	BOMV	MARV
Anti-EBOV	++++	+	++	++	++	+	+
Anti-SUDV	–	+++	–	–	–	–	–
Anti-RESTV	–	–	++++	–	–	–	–
Anti-BDBV	+	–	+	++++	+	–	–
Anti-TAFV	–	–	–	+	+++	–	–
Anti-BOMV	–	–	–	–	+	++++	–
Anti-MARV	–	–	–	–	–	–	+++

Serum samples were first screened in duplicate for reactivity against the recombinant full-length EBOV-GP protein (0.5μg/ul). Sera were heat inactivated in a water bath at 60 °C for 30 min. Microtiter plates (Invitrogen, USA) were coated overnight (4 °C) with 25 ng/well of EBOV rGP, blocked with 3% goat serum and incubated with 1:200 dilution of heat-inactivated test sera. Antibody binding was detected by HRP conjugated anti-human IgG (1:10000, Seracare Life Sciences Inc., Milford, MA USA), followed by o-phenylenediamine dihydrochloride (OPD) and stopped with 1 M sulphuric acid. Optical densities (OD) were read at 490 nm (μQuant™ - BioTek). Controls included a polyclonal serum (1:2000) raised in rabbits against the EBOV rGP (eEnzyme LLC) with binding detected by HRP conjugated anti-rabbit IgG (1:5000, ImmunoReagents, Raleigh, NC USA), EBOV seropositive and seronegative human serum samples (1:200) from Uganda [13], and commercially available negative human serum (1:200, Millipore Sigma, Burlington, MA USA). A sample was considered reactive when the absorption was higher than 3 times the background (no antigen) or the negative wells (whichever was higher). Because of the potential for cross-reactivity, EBOV-reactive samples were then screened (1:200) against the rGP of all six ebolaviruses (EBOV 0.25μg/ml, SUDV 0.25μg/ml, BDBV 0.25μg/ml, RESTV 0.25μg/ml, TAFV 4μg/ml, BOMV 0.25μg/ml) and MARV (0.25μg/ml) to see if the reactive sera showed stronger reactivity to a different filovirus. Positive controls included polyclonal antibodies raised in rabbits against the EBOV (1:2000), SUDV (1:1000), BDBV (1:2000), RESTV (1:2000, TAFV (1:1000), BOMV (1:2000) and MARV (1:2000) rGPs. Finally the endpoint titer of positive samples was determined by two-fold dilutions using the antigen to which the strongest reactivity was observed.

Western blotting was used to confirm ELISA-positive samples. Briefly, 50 ng (for use with polyclonal rabbit sera) and 250 ng (for use with human sera) of EBOV-rGP (R&D systems) and BOMV-GP were separated under denaturing conditions on Bolt™ 4–12% Bis-Tris Plus Gels (Invitrogen, USA) and transferred to Low-Fluorescence PVDF transfer membranes (Invitrogen, USA). Membranes were blocked with 5% non-fat milk and 1% goat serum (1 h at RT) before adding heat-inactivated serum (1:200) and positive (polyclonal antibody raised in rabbits, 1:5000, eEnzyme) and negative (1:200, negative pateint serum) controls and incubated at RT overnight. Antibody was detected with either peroxidase-labeled goat anti-human IgG (1:10000, Seracare, MA) or goat anti-rabbit IgG (1:5000 ImmunoReagents). Signal was detected using Supersignal West Pico PLUS chemiluminescent substrate (Invitrogen, USA) on the iBright Imager (Invitrogen). Blots were considered positive if a band of the correct size was visualized for the GP_1_ protein (~ 120 kDa).

A peptide microarray based on the Roche Nimblegen platform [24, 25] was also used to support the detection of antibodies to ebolaviruses. The Serochip array included 63,281 16-mer linear overlapping peptides with an offset of four amino acids covering the six ebolavirus and MARV (RAVV, species *Marburg virus*) proteomes (Genbank accession numbers NC002549, NC004161, NC006432, NC014372, NC014373, NC024781, MC039345). The peptides are printed in random positions on the peptide array to minimize the impact of locational bias. A subset of serum samples that were positive for antibodies to EBOV (*n* = 5, titers ranging from 1:100 to 1:12,800) and BOMV (*n* = 1, 1:800) by ELISA and a commercially available negative human serum were tested on the Serochip at 1:50 dilution, as described previosly [26, 27]. Polyclonal sera raised in rabbits against the GP of all six ebolaviruses and MARV were also tested on the Serochip at 1:50 dilution for comparison to test sera. A reactive epitope was identified by a continuous set of overlapping 16-mer peptides (two or more) with signal intensities above a threshold of 10,000-AU [27]. All immunoreactive linear epitopes were identified as ebolavirus through tblastn against the GenBank protein database. The number of identified ebolavirus epitopes was tallied by gene in human and polyclonal rabbit serum samples and the immunoreactivity of epitopes compared with each other and that of negative controls.

### Data analysis

Differences in seropositivity by age and sex were evaluated using the χ2 test. Generalized linear models were used to evaluate associations between EBOV seropositive status and factors such as age, sex, and clinical symptoms. Odds ratios were then calculated for significant associations.

All statistical analyses were performed using R (R Foundation for Statistical Computing Vienna, Austria) [28].

## Results

Samples were collected and tested from 272 people seeking care in the Rutshuru Health Zone in the DRC between May 2017 and April 2018. The Rutshuru Health Zone is to the south of the Mabalako Health Zone in North Kivu where the 2018–2020 EVD outbreak began in July 2018 (Fig. 1). Participants traveled from the villages of Rubare (*n* = 181), Ntamugemga (*n* = 73), Kiwanja Umoja (*n* = 6), Biruma (*n* = 5), Kalengera (*n* = 6) and Rugari (*n* = 1); ranged in age from 2 to 68 years and included 120 children (2–17 yrs) and 152 adults (18+ yrs); and identified as males (*n* = 108) and females (*n* = 164) of all ages. Participants presented to the clinic with fever, and a range of other clinical symptoms including headache (*n* = 240), malaise (*n* = 182), lack of appetite (*n* = 169), chills (169), and vomiting (*n* = 146). Other less common clinical symptoms included abdominal pain, joint pain, dark urine, altered consciousness and bleeding. None were diagnosed with hemorrhagic fever at the time of treatment or had been previously diagnosed with EBOV infection or EVD. Livelihoods mainly consisted of crop production, growing a combination of fruit, vegetable, coffee, tea and cocoa crops (*n* = 118); and students (*n* = 122). Limited contact was reported with wild animals, such as bats (*n* = 5), non-human primates (*n* = 6), and wild ungulates (*n* = 2), but contact with rodents (*n* = 153) and domestic animals [mostly poultry (*n* = 226), goats and sheep (*n* = 141), and dogs (*n* = 44)] was common. Animal contact was primarily through raising and handling animals, having animals kept in the house, and consumption of meat, mainly poultry, sheep, or goats. None of the patients enrolled in the study reported travelling outside the area.

**Fig. 2:**
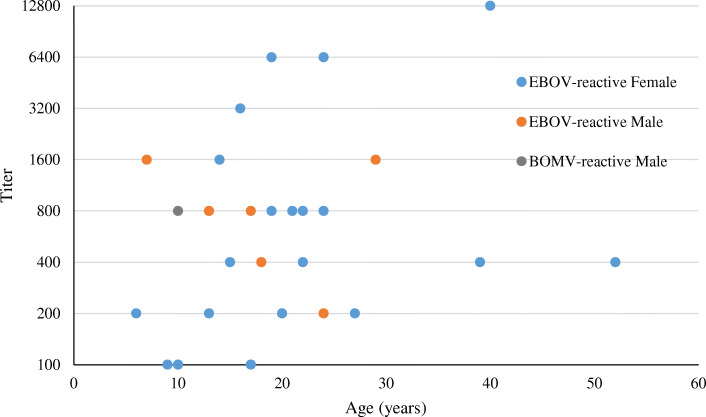
Distribution of titers detected by ELISA to EBOV and BOMV by sex and age

Serum, oral swab and fecal samples from all patients were negative for filoviruses by all four PCR assays. Seven patients were PCR postive for other viruses, five for Human Coronavirus OC43 and two for Influenza A. Initial screening by indirect ELISA found that 30 of the 272 people were reactive to the EBOV-rGP (Table 2). Differential screening of all reactive serum samples against the rGP of all six ebolaviruses and MARV showed that 29 people exhibited the strongest reactivity to EBOV and one to BOMV. Serial dilution to determine the end-point titers showed that titers to EBOV ranged from 1:100 to 1:12,800 (Fig. 2) and the titer in the BOMV positive person was 1:800. Despite proximity to regions in Uganda where outbreaks of BDBV and SUDV have been reported, there was no evidence of exposure to these viruses in our cohort. One patient with a low titer to EBOV (1:100) was also PCR positive for Human Coronavirus OC43, all other patients that were PCR positive for Human Coronavirus OC43 or Influenza A were negative for antibodies to ebolaviruses.

**Table 2:** Seroprevalence to ebolaviruses by village and sex

Village	Positives			
	Female	Male	Total tested	Proportion Positive
Biruma	0	0	5	0
Kalengera	0	0	6	0
Kiwanja Umoja	1	0	6	16.7
Ntamugemga	3	2	73	6.8
Rubare	19	5^a^	181	12.7
Rugari	0	0	1	0

^a^1/5BOMV positive

**Fig. 3:**
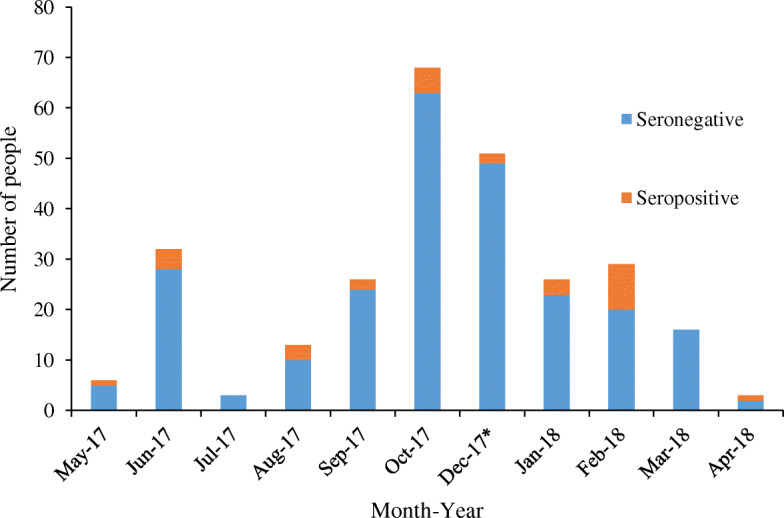
**a.** EBOV Western blot. Lane 1 = Rabbit anti-EBOV GP polyclonal serum, Lane 2 = EBOV positive patient serum, Lane 3 = Negative patient serum, Lane 4 = BOMV positive patient serum. **b.** BOMV Western blot. Lane 5 = Rabbit anti-BOMV GP polyclonal serum, Lane 6 = BOMV positive patient serum, Lane 7 = Negative patient serum, Lane 8 = EBOV positive patient serum. L = molecular weight ladder. GP1 band is approximately at 120 kDa

Screening results from the indirect ELISA were supported by detection of ebolavirus antibodies using the Serochip platform. We first identified immunoreactive linear peptides in the GP using polyclonal rabbit sera raised against all of the ebolaviruses, but they could not be used to discriminate between the ebolaviruses (Supplemental Table 1). We also identified reactive epitopes as belonging to ebolaviruses by comparing against the Genbank protein database in all six human samples. In the EBOV seroreactive patient with the highest titer (1:12,800) we identified nine reactive epitopes in four genes in an EBOV seroreactive patient and 52 reactive epitopes in six genes in the BOMV seroreactive patient (titer 1:800) (Table 3). However, reactive epitopes did not discriminate between EBOV and BOMV reactive samples, as cross reactivity occurred in all samples against all six ebolaviruses and MARV. We did detect reactivity to a small number of peptides in the negative serum sample (Table 3) indicating that there was potentially some cross reactivity to proteins from other unknown antigens. Instead the differential detection of antibodies to EBOV and BOMV were confirmed by western blot. The patient sera presumptively positive for antibodies to EBOV bound to EBOV rGP and the patient serum presumptively positive for antibodies to BOMV bound to BOMV rGP, but not vice versa (Fig. 3).

**Table 3:** Number of immunoreactive linear epitopes identified in human EBOV (1:12,800) and BOMV (1:800) reactive serum samples compared to a negative human serum sample by gene and overall for all six ebolaviruses and Marburg virus

	EBOV Presumptive Positive Patient								BOMV Presumptive Positive Patient								Negative Human							
Peptide	NP	VP35	VP40	GP	VP30	VP24	L	Tot	NP	VP35	VP40	GP	VP30	VP24	L	Tot	NP	VP35	VP40	GP	VP30	VP24	L	Tot
EBOV	2		1	3			3	**9**	9		4	10	4		16	**43**								
BOMV	4			2	1		4	**11**	10	6	3	10	3	2	18	**52**		1		1			1	**3**
SUDV				1		1	2	**4**	8		1	12	5		16	**42**				1			1	**2**
BDBV	2			3	1		4	**10**	7		1	10	2		15	**35**								
TAFV	3				1		2	**6**	9		3	10	2		18	**42**								
REST	3			2			4	**9**	9			11	3		18	**41**	2			3				**5**
MARV	2						1	**3**	9		4	13	3		22	**51**				2	1			**3**

**Fig. 4:**
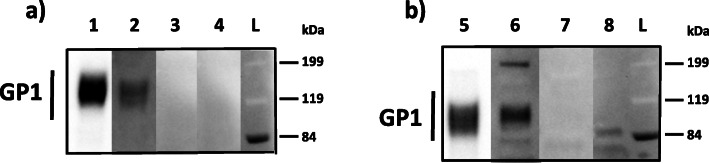
Distribution of EBOV and BOMV (*) seropositive samples by month and year by indirect ELISA

Most of the seropositve people for EBOV were from Rubare village (22 out of the 29 positives) and the one BOMV positive person was also from Rubare, a 10-year old male child. EBOV positives ranged in age from 6 to 52 years. Proportionally more females were positive than males (*p* = 0.04) and females were significantly more likely to be seropositive than males (OR = 2.8, 95% CI 1.2 to 7.7). Equal proportions of adults (16 out of 152 or 10.5%) and children (13 out of 120 or 10.8%) were seropositive (*p* = 1). The four highest positive titers were from females and the two oldest people with the highest titers were also female. Seropositive samples were collected in almost all study months, with the majority of EBOV positives (12/29) detected in patients sampled in February 2018 (Fig. 4), the single BOMV seropositive sample was collected in December 2017. The most common clinical symptoms reported in seropositive people were fever, headache, malaise (fatigue), vomiting, chills, altered consciousness, abdominal pain, dark urine, joint pain, and bleeding, however, these symptoms were also present in seronegative people. Relative risk among the symptoms was the greatest for dark urine (present in 31% of EBOV positive people and 14% of EBOV negative people; and those with dark urine were more likely to be EBOV positive than patients that did not report dark urine (OR = 3.01, 95% CI 1.2 to 7.1). There was no association with crop production as a livelihood in seropositive individuals. Finally, limited contact with wildlife was reported, so association with seropositivity could not be assessed, and contact with domestic species was common with no association with seropositivity.

## Discussion

We provide the first evidence of exposure to Ebola virus in people in eastern DRC as we detected antibodies in 10% of febrile patients seeking care prior to recogniton and declaration of the 2018–2020 EVD outbreak. However, our results did not link any specific clinical symptoms with the presence of ebolavirus antibodies. Increased contact between humans and wildlife has been linked to spillover and precipitating EVD outbreaks [29–31]. It is possible that spillover occurred much earlier into people in eastern DRC and that EBOV was circulating much longer before recognition of associated illness. Alternatively, our data may suggest that spillover from wild animals into humans occurs more frequently than is recognized, and may not always precede or precipitate EVD outbreaks. These results are also the first documentation of antibodies against BOMV in a person and thus provide early evidence that spillover from this virus from bats to humans has likely occurred. The BOMV seropositive patient was a child who presented with a fever, headaches and severe fatigue, but who was negative for BOMV by PCR. Therefore, it is unknown if BOMV was associated with illness or if exposure was purely incidental.

The serologic assays performed were designed to detect antibodies to IgG, so we cannot determine how recently exposure occurred. Since the majority of EBOV antibody-positive patients were detected in February 2018, it is possible that we detected early paucisymptomatic Ebola virus cases before recognition of severe EVD associated with the outbreak. Given that we detected antibodies in people as early as May 2017, our data also support analysis suggesting that EBOV could have been circulating in the North Kivu region 1–2 years before the outbreak began [5]. It is not known how frequently asymptomatic or paucisymptomatic Ebola virus infection occurs, however, a number of studies show a high level of exposure in asymptomatic household contacts of EVD-positive patients, and in contacts with mild clinical symptoms who were not diagnosed with EVD [32, 33]. EVD symptoms can vary from flu-like to acute hemorrhagic fever and death [34], thus, the non-specific nature of some clinical symptoms may mean that EVD cases are not recognized until more severe symptoms (eg. hemorrhagic disease), increased level of exposure, and deaths occur. A range of common non-specific clinical symptoms were reported in the majority of people seeking care in this study. However, some less common clinical symptoms such as, dark urine, altered consciousness, and bleeding, were also reported. The presence of dark urine (perhaps suggestive of the presence of blood in the genitourinary tract) was significantly associated with the detection of antibodies to EBOV. Given that long term immunity has been documented in survivors [35], it is probable that we detected a combination of previous infection and recent paucisymptomatic illness. If we detected undiagnosed paucisymptomatic illness prior to the current outbreak, our results support previous studies with the important recognition that ebolaviruses likely cause disease with a range of severity, including mild unrecognized illness in people [36, 37].

Although both sexes and all ages tested positive for antibodies, women were significantly more likely to be positive and 45% of the positives were children, including the one BOMV positive child. These results are consistent with other studies as women have been shown to have an increased risk for exposure and prevalence of infection, likely due to their gender roles and responsibility of caring for children, the sick and for animals [38, 39]. Similarly, children have had high seroprevalence in other parts of Africa and misdiagnosis could explain the apparent lower incidence of EVD in children [32, 40]. Children are likely coming into contact with wild animals more frequently through hunting or play, and are therefore at higher risk for exposure to ebolaviruses. Indeed the index case of the 2013 outbreak in West Africa is believed to have been a 2-year old child that had contact with Angolan free-tailed insectivorous bats (species: *Mops condylurus*) prior to becoming ill [41]. Previous work has shown that people in rural areas living in close proximity to forests, with exposure to rodents, duikers, non-human primates and bats through and hunting and eating have antibodies to ebolaviruses [42–44].

We developed and used a combination of serologic assays to screen for and discriminate between antibodies to ebolaviruses. Both the initial screening with the indirect ELISA and Serochip assays were able to identify seroreactive patients but could not discriminate between antibody responses to the ebolaviruses. The serochip data did support reactivity to multiple epitopes in multiple genes rather than just one based on the ELISA assay. The differential ELISA screening was able to discern which rGP had the strongest reactivity in each sample and western blotting was able to confirm the antibody response to EBOV and BOMV. Titers measured were as high as 1:12,800 and were as high as or higher than those measured (by other assays) in survivors [45].

## Conclusions

In conclusion, our study provides evidence of spillover of ebolaviruses into people prior to recognition of disease in febrile patients seeking care in North Kivu Province. While our study does not confirm that ebolaviruses were the cause of the observed clinical signs, we found antibodies to Ebola virus in febrile people prior to the start of the 2018–2020 EVD outbreak, suggesting early cases could have been missed or that exposure ocurred without associated severe illness. We also document the first detection of antibodies to Bombali ebolavirus in a person and show that spillover of BOMV from bats to humans has likely occurred. Our data support growing evidence that a range of severity of EBOD illness occurs in people and that human exposure is more frequent and geographically widespread.

## Supplementary information

**Additional file 1: Supplemental Table 1.** Number of immunoreactive linear epitopes identified in polyclonal rabbit sera raised against the rGP of all six ebolaviruses by gene and overall for all six ebolaviruses.

## Data Availability

The datasets during and/or analysed during the current study available from the corresponding author on reasonable request.
