# Supplementary material for: Spillover of ebolaviruses into people in eastern Democratic Republic of Congo prior to the 2018 Ebola virus disease outbreak
Source: One Health Outlook. 2020 Nov 4;2:21. doi: 10.1186/s42522-020-00028-1 (PMC7609368; doi:10.1186/s42522-020-00028-1)
Supplement: Supplementary file 1 — Additional file 1: Supplemental Table 1. Number of immunoreactive linear epitopes identified in polyclonal rabbit sera raised against the rGP of all six ebolaviruses by gene and overall for all six ebolaviruses. [file 42522_2020_28_MOESM1_ESM.docx]

**Supplemental Table 1.** Number of immunoreactive linear epitopes identified in polyclonal rabbit sera raised against the rGP of all six ebolaviruses by gene and overall for all six ebolaviruses.

|  | **Anti-EBOV GP Rabbit** | | | | | | | | | **Anti-BOMV GP Rabbit** | | | | | | | | **Negative Human** | | | | | | | |
| --- | --- | --- | --- | --- | --- | --- | --- | --- | --- | --- | --- | --- | --- | --- | --- | --- | --- | --- | --- | --- | --- | --- | --- | --- | --- |
| **Peptide** | **NP** | | **VP35** | **VP40** | **GP** | **VP30** | **VP24** | **L** | **Tot** | **NP** | **VP35** | **VP40** | **GP** | **VP30** | **VP24** | **L** | **Tot** | **NP** | **VP35** | **VP40** | **GP** | **VP30** | **VP24** | **L** | **Tot** |
| EBOV | 1 |  | |  | 12 |  |  | 1 | **14** |  |  | 1 | 7 |  |  | 3 | **11** |  |  |  |  |  | 1 |  | **1** |
| BOMV |  |  | |  | 9 |  |  | 1 | **10** |  |  |  | 13 |  | 1 |  | **14** |  |  |  |  |  |  |  |  |
| SUDV |  |  | |  | 9 | 1 |  | 1 | **11** | 1 |  |  | 9 |  |  | 2 | **12** |  |  |  | 1 |  |  | 2 | **3** |
| BDBV | 2 |  | |  | 9 |  |  |  | **11** | 3 |  |  | 9 |  |  | 1 | **13** |  |  |  |  |  |  |  |  |
| TAFV |  |  | |  | 13 |  |  | 2 | **15** | 1 |  |  | 9 |  |  | 1 | **11** |  |  |  |  |  |  |  |  |
| REST |  |  | | 1 | 6 |  |  | 3 | **10** | 2 |  |  | 6 |  |  |  | **8** |  |  | 1 | 1 |  |  | 2 | **4** |
